# A Swiss Retrospective Case Series of Pediatric Primary Subacute Hematogenous Osteomyelitis

**DOI:** 10.3390/microorganisms14020514

**Published:** 2026-02-23

**Authors:** Elio Paris, Giacomo De Marco, Ahmer A. Khan, Anne Tabard-Fougère, Oscar Vazquez, Christina Steiger, Romain Dayer, Dimitri Ceroni

**Affiliations:** 1 Faculty of Medicine, University of Geneva, 1211 Geneva, Switzerland; 2 Paediatric Orthopedics Unit, Geneva University Hospitals, University of Geneva, 1205 Geneva, Switzerland

**Keywords:** subacute, hematogenous, osteomyelitis, etiology, microbiology, *Kingella kingae*, nucleic acid amplification test, magnetic resonance imaging

## Abstract

This study aimed to characterize the clinical spectrum and the bacteriological and microbiological etiology of pediatric primary subacute hematogenous osteomyelitis (PSAHO) and to evaluate a modern diagnostic approach for these infections. A single-center, 25-year retrospective review (2000–2025) of 107 consecutive cases of PSAHO was performed. Clinical presentation, traditional inflammatory markers, conventional cultures and nucleic acid amplification tests (NAATs) on blood, and bone and oropharyngeal samples were assessed. Most patients (73.8%) were <4 years. Fever was uncommon (15.9%), and inflammatory markers were frequently normal (white blood cell counts in 81.1%, C-reactive protein levels in 60.4%) and abnormal in 69.2% for erythrocyte sedimentation rates and in 53.8% for platelet count. Low diagnostic sensitivity of conventional blood (4.2%) and bone (25.7%) sample cultures has been reported. In contrast, a comprehensive NAAT-based approach identified or strongly suggested a pathogen in 44.9% of cases. *Kingella kingae* was the predominant pathogen in children under 4. Oropharyngeal PCR tests potentially identified the pathogen in another 20 cases, and its presence could be reasonably suspected in a further 68 (63.6%). MRI was essential for diagnosis, identifying all lesions, whereas the sensitivity of radiographs was low (<50%). All patients recovered completely, regardless of the management strategy. This study provides three critical advances for clinical practice: (1) PSAHO should be considered in a limping toddler even without fever or elevated inflammatory markers, and MRI is the imaging modality of choice; (2) NAATs are indispensable for etiologic diagnosis, revealing age-dependent pathogens; (3) Oropharyngeal PCR is a useful diagnostic adjunct.

## 1. Introduction

Primary subacute hematogenous osteomyelitis (PSAHO) is an infectious process affecting bones that is characterized by localized bone pain, mild or no systemic manifestations, non-contributory laboratory results, and blood cultures, but positive radiological findings [1,2,3,4,5,6,7,8,9,10,11,12]. According to King and Mayo, any osseous infection without acute symptomatology lasting more than 2 weeks but less than 3 months is referred to as subacute osteomyelitis [1]. Thus, several entities coexist under this vague definition, with the only common denominator being the diagnostic delay leading to lytic lesions. Forms of subacute hematogenous osteomyelitis are usually linked to host-pathogen relationships. This can be conjugated in different ways, including decreasing the virulence of the causative organism, increasing host resistance, and early exposure to antibiotics [3,6,7,12,13]. PSAHO occurs mainly in children and must be distinguished from osteomyelitis due to other conditions, such as chronic recurrent multifocal osteomyelitis or SAPHO syndrome (synovitis, acne, pustulosis, hyperostosis, and osteitis) [13]. Standard bone sample cultures usually fail to identify the causative organism, resulting in negative cultures in 25–60% of patients [12]. Since the early 2000s, the widespread use of nucleic acid amplification tests (NAATs) has significantly improved the identification of the pathogens responsible for osteoarticular infections [14], including when applied to PSAHO [12]. Indeed, NAATs have highlighted the role played in these infections by less virulent and fastidious pathogens, such as *Kingella kingae* (*K. kingae*). When considering children’s ages and their infections’ bacteriological etiologies, these studies suggested the presence of two forms of PSAOH [12]. The first form—the infantile form—affects children aged from 6 months to <4 years and is mainly caused by *K. kingae*. The second form—the juvenile form—involves children > 4 years, and the main bacteriological etiology is *Staphylococcus aureus* (*S. aureus*). The present study aimed to describe the spectrum of cases of PSAHO found among children, to characterize their clinical presentation and biological features, and to investigate their bacteriological etiology. It also aimed to demonstrate how NAATs have contributed to our growing understanding of PSAHO and how early use of magnetic resonance imaging (MRI) can shorten the diagnostic delay and prevent the onset of lytic lesions in bones.

## 2. Materials and Methods

### 2.1. Studied Population

Consecutive pediatric patients aged from 1 day to 16 years, admitted to our facility with PSAHO between January 2000 and June 2025, were retrospectively identified. Diagnosis codes for osteomyelitis, septic arthritis with concomitant osteomyelitis, and spondylodiscitis were used to identify potential cases of interest in our institution’s electronic medical records. To be eligible for enrollment in the present study, patients had to have sustained an osseous infectious process lasting more than 2 weeks and less than 3 months and to have presented with a lytic lesion confirmed by radiological methods. Eligibility was determined by radiographic evidence, and the presence of mild laboratory disturbances (white blood cell count (WBC), erythrocyte sedimentation rate (ESR), and serum C-reactive protein level (CRP)) was not an exclusion criterion. This study was approved by the cantonal ethics committee (2023-00578).

### 2.2. Epidemiological, Clinical, and Paraclinical Investigations

Data collected included epidemiological data on age, sex, and the bones involved. Clinical and paraclinical investigations included rectal temperature at admission and laboratory values, such as WBC count, platelet count, ESR, and CRP level.

### 2.3. Microbiological Work-Up

Blood cultures were performed almost systematically. When taken, bone aspirate samples were sent to the laboratory for immediate inoculation. Since 2007, broad-range polymerase chain reaction (PCR) assays and *K. kingae*-specific real-time PCR assays have been performed on bone aspirate samples for the identification of causative organisms. Since September 2009, oropharyngeal swab PCRs were performed for children from 6 months to <4 years old, since it has been shown that detecting *K. kingae* rtx toxin genes in the children’s oropharynx provided strong evidence that this microorganism was responsible for an osteoarticular infection, or even stronger evidence that it was not [15].

### 2.4. Imaging Investigations

Plain radiographs were available for all patients; when accessible, MRI was performed before or within 48 h of admission [16,17]. Images were acquired (i) at 1.5-T (Avanto; Siemens) using tridimensional Short Tau Inversion Recovery (STIR) with T1-weighted turbo spin-echo (one longitudinal plane), (ii) in two orthogonal planes using T2-weighting and fat suppression, STIR (longitudinal plane), and water-only fast spin-echo T2-weighted Dixon sequences (axial plane), and (iii) using diffusion weighted imaging (axial plane) and post-contrast injection T1-weighted spin echo with frequency-selective fat saturation (in two orthogonal planes). Postcontrast sequences were obtained after the injection of 0.2 mL/kg of gadoteric acid (Dotarem).

### 2.5. Sample Size Estimation

This study is a retrospective analysis of a complete cohort; therefore, no prospective sample size calculation was performed. All patients meeting the diagnostic criteria for PSAHO during the 25-year study period were included to minimize selection bias and maximize generalizability.

The cohort of 107 consecutive patients is one of the largest single-center studies on PSAHO and provides robust data for analyzing demographic, clinical, and biological characteristics. For our key finding—that PCR-based methods identified a pathogen in 44.9% (*n* = 48/107) of cases—the 95% confidence interval (calculated using the Wilson score method) is 35.5%–54.6%. This provides a clinically meaningful level of precision for estimating the true diagnostic yield of advanced microbiological techniques in this population.

### 2.6. Statistical Analysis

All analyses were performed using R software (version 4.4.1, R Foundation for Statistical Computing, Vienna, Austria) and the RStudio interface (version 2024.09.0, Posit software, PBC). The level of significance was set at *p*-values < 0.05. The normality of the data distribution was assessed using the Shapiro–Wilk test. The characteristics of patients with PSAHO were initially analyzed considering all the patients together and then by defining two diagnostic subgroups according to age (<4 and ≥4 years). The normality of the distributions of the patients’ clinical manifestations and laboratory test results was evaluated using the normal Q-Q plot and the Shapiro–Wilk test. Comparisons between patients < 4 years and ≥4 years were performed using an unpaired Mann–Whitney *U* test, with values reported as median and interquartile range (IQR). Patients were then separated into three groups based on their causative germs: (1) *K. kingae* (confirmed + suspected), (2) no pathogen identified, and (3) other pathogens (e.g., *methicillin-susceptible Staphylococcus aureus [MSSA]*, *Staphylococcus speciae*, and *Streptococcus speciae*). These three groups were compared using the Kruskal–Wallis test, and post hoc outcomes were evaluated using unpaired Mann–Whitney–Wilcoxon tests, with an adjustment of post hoc *p* values, multiplying them by 3 (the number of post hoc calculations).

## 3. Results

### 3.1. Epidemiology, Skeletal Distribution, and Clinical Characteristics

**Figure 1:**
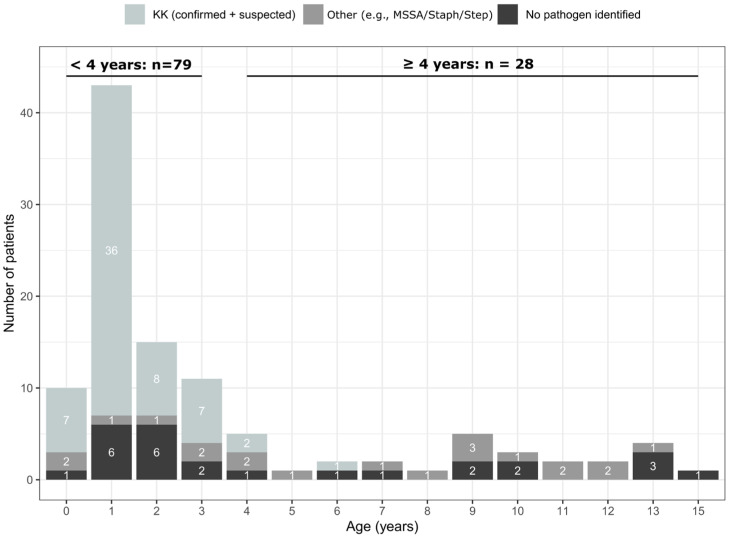
Distribution of patients with subacute osteomyelitis considering their age and the causative agents. To do this, patients were separated into three groups based on their causative germs: (1) *K. kingae* (confirmed + suspected), (2) no pathogen identified, and (3) other pathogens (e.g., *methicillin-susceptible Staphylococcus aureus* [MSSA], *Staphylococcus speciae, Streptococcus speciae*).

This study included 107 children (52 girls, 55 boys) with PSAHO. The mean age was 46.5 ± 45.9 months, with a clear predominance (79/107, 73.8%) in children under 4 years; the largest subgroup was those of children aged 13–24 months (43/107, 40.2%), and no cases under 6 months of age were encountered (Figure 1).

**Figure 2:**
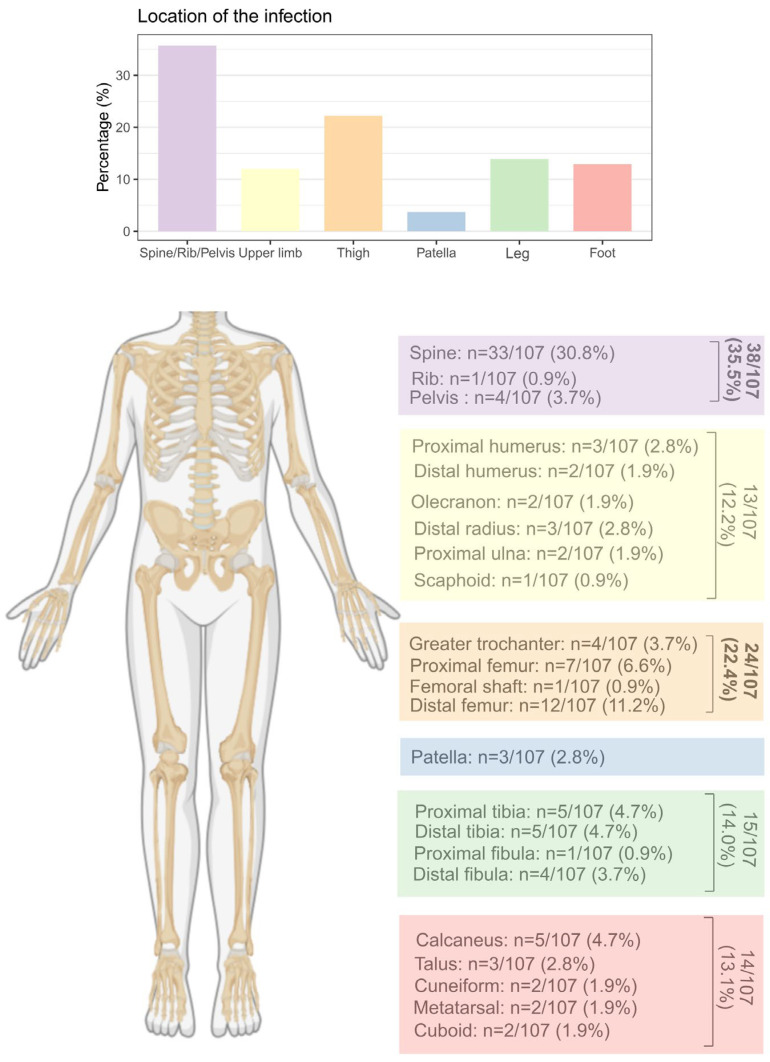
Localization of subacute osteomyelitis. purple = spine/rib/pelvis; yellow = upper limb; orange = thigh; blue = patella; green = leg; red = foot.

The most common infection sites were long bones (53/107, 49.5%) and spine (34/107, 31.8%) (Figure 2). Only two patients had prior exposure to antibiotics (<48 h before). Functional impairment was the universal presenting symptom, while fever was rare (17/107, 15.9%).

### 3.2. Inflammatory Markers

As reported in Table 1, normal values were evaluated for WBC count (<12,000 cells/μL) in 86/106 cases (81.1%) and for CRP (<10 mg/L) in 64/106 cases (60.4%). When performed (91 cases), the ESR was greater than 20 mm/h in 63 cases (69.2%), and 57 children (53.8%) had abnormal platelet counts (>400,000 platelets/μL).

When all four temperature and blood inflammatory markers were available (91 cases), WBC, ESR, CRP, and rectal temperature were all within normal ranges in 21 cases (23%). A single marker was abnormal in 30 children (33%), two were abnormal in 27 cases (29.7%), and three markers were abnormal in 13 cases (14.3%). There were no cases where all four markers were abnormal (Table 2).

### 3.3. Comparisons of Clinical and Biological Data by Age

**Figure 3:**
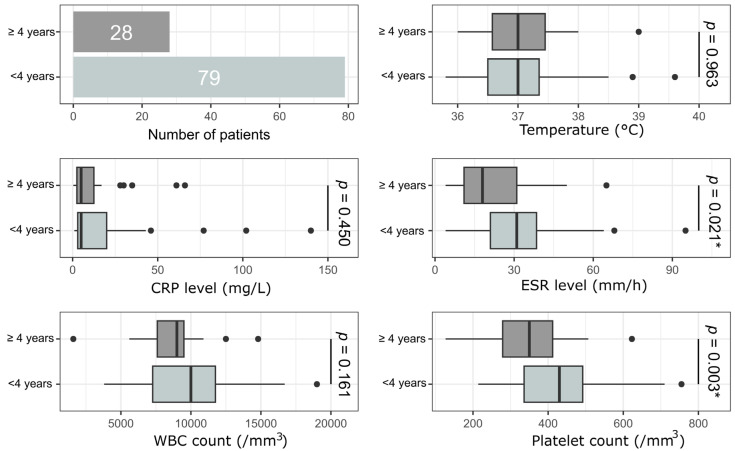
Comparative analysis of clinical and biological parameters based on patient age (<4 and ≥4 years). Comparisons between groups were performed using an unpaired Mann–Whitney U test. CRP is C-reactive protein, WBC is white blood cell, and ESR is erythrocyte sedimentation rate. *: *p* < 0.05.

Our first interest was whether PSAHO’s clinical and biological presentation varied between children < 4 years old and older children; there were significant differences between the two groups’ ESR and platelet count values (Figure 3).

### 3.4. Comparisons of Clinical and Biological Data Considering Pathogens

**Figure 4:**
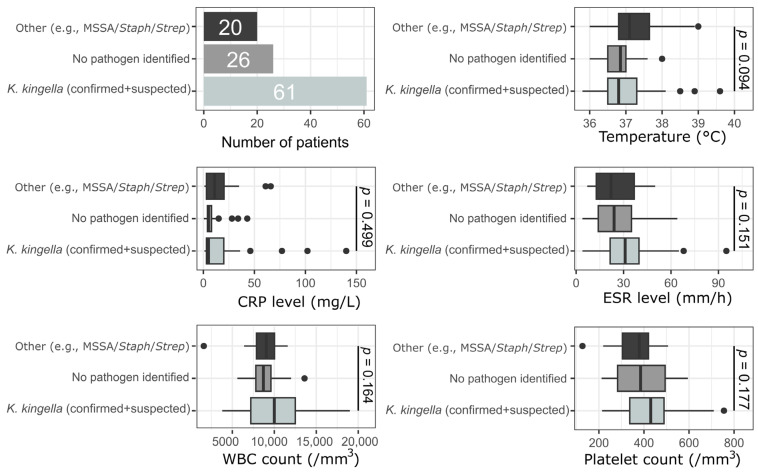
Comparative analysis of clinical and biological parameters by the germ responsible for the subacute osteomyelitis. Patients were separated into three groups based on their causative germs: (1) *K. kingae* (confirmed + suspected), (2) no pathogen identified, and (3) other pathogens (e.g., *methicillin-susceptible Staphylococcus aureus* [MSSA], *Staphylococcus speciae*, and *Streptococcus speciae*). Groups were compared using the Kruskal–Wallis test, and post hoc outcomes were evaluated using unpaired Mann–Whitney–Wilcoxon tests, with an adjustment of post hoc p values. CRP is C-reactive protein, WBC is white blood cell, and ESR is erythrocyte sedimentation rate.

Our second interest was knowing whether the pathogen responsible for PSAHO could lead to sufficiently significant differences in children’s clinical and biological presentation at admission. We noted no significant differences in the children’s clinical and biological parameters depending on the microorganisms detected (Figure 4). Finally, analysis between patients with microbiologically confirmed *K. kingae* infection and those with only suspected infection demonstrated no significant differences in clinical and laboratory parameters.

### 3.5. Bacteriological Investigations

Blood cultures were obtained from 72 children and identified a pathogen in three patients (3/72; 4.2%) (Table 3). Aerobic or anaerobic bacterial cultures of bone material were performed for 70 patients. Thirty-seven children underwent neither open nor percutaneous surgical drainage (essentially in cases of spondylodiscitis). In 18 cases, the pathogen was isolated from bone samples using classic culture methods (25.7%). The use of PCR assays from 2007 to 2025 enabled the causative microorganism to be isolated from bone aspirates in an additional 26 cases among 33 patients (78.8%). Blood PCR analysis was performed in 17 patients with spondylodiscitis, and three positive cases were detected (17.7%). Using broad-range PCR on a sample of 15 patients, we identified 2 positive cases (13.3%) (one *MSSA* and one *Streptococcus pneumoniae*). Examining the results from classic cultures of blood and bone samples, a pathogen was detected in 19 cases (17.8% of children investigated bacteriologically). A pathogen was detected in 48 cases that used the results from blood cultures, classic cultures, and different PCR assays (44.9% of 107 patients). Finally, 20 children were positive for *K. kingae* DNA in their oropharyngeal swabs, despite undergoing no other investigations and having only a negative blood culture. These 20 cases, therefore, were highly suggestive of a *K. kingae*-induced PSAHO and could potentially be added to the list of patients in whom a pathogen was identified. The presence of a pathogen could thus reasonably be suggested in a total of 68 cases (63.6% of children).

In no case were multiple organisms involved. In addition, we did not encounter cases of discordant microbiological findings between culture and PCR results from the same site or within the same patient.

The PSAHO pathogens identified are listed in Table 4 and classified according to patient age in Figure 1 and Figure 4.

### 3.6. Radiological Investigations

Complete radiological records were available for 96 children (89.7%), including both X-rays and MRI scans. Conventional X-rays detected PSAOH lesions in 46 cases (47.9%); MRI detected them in every case.

### 3.7. Treatment

During initial care, 32 cases of PSAHO were percutaneously aspirated, 39 required open debridement and curettage of the lesion, and 36 were treated without surgical procedure. The initial treatment choice was made by the admitting orthopedic surgeon; in all cases, intravenous treatment started 24 h after the samples were collected. The antibiotic treatment was then switched to oral therapy, adapted to the pathogens found. All patients, whether treated surgically or non-surgically, received antibiotics for 3–4 weeks. For methicillin-susceptible strains (MSSA), resistant penicillin, such as amoxicillin-clavulanic acid, has been used. Treatment of both S. pyogenes and S. pneumoniae was primarily based on amoxicillin or penicillin V. Since no specific protocols for managing invasive *K. kingae* OAIs have been dictated, the empirical drug regimen was generally based on a penicillinase-stable β-lactam antibiotic (such as amoxicillin-clavulanic acid) or on broad-spectrum second-generation (cefuroxime) cephalosporins. All patients responded well to these regimens, with no cases of treatment failure or a recurrence of infection. Only one patient required a segmental resection of the infected bone, followed by a bone reconstruction. Visible lytic lesions disappeared in every case, and healing was evident in radiographs within 3–12 months.

## 4. Discussion

Despite improvements in medical imaging and bacteriological investigation techniques, PSAHO remains a distinct form of osteomyelitis that is difficult to diagnose because of its insidious onset, mild symptoms, and the inconsistency of supportive laboratory data. Accurate diagnosis is usually significantly delayed, therefore, often until after a lytic bone lesion becomes visible on plain radiographs, and thus, PSAHO remains a poorly recognized disease. To the best of our knowledge, this work reports on the largest series ever published of cases of PSAHO with MRI evaluations. It provides very interesting new information that complements the existing epidemiological data.

First, our findings showed that cases of PSAHO occurred at a higher rate than previously reported. The cases of PSAHO in this series represent 30.7% of all patients with hematogenous osteomyelitis at our institution. Interestingly, the same observation was made by Jones et al., who reported a subacute osteomyelitis rate of 35% and concluded that this type of bone infection was becoming more widespread [8]. A more recent study confirmed this frequency by evaluating the annual incidence of subacute osteomyelitis at 5 per 100,000 in the pediatric population [18].

The present study also confirmed that PSAHO must be described in two different forms, closely correlated to patient age and bacteriological etiology [12]. The infantile form is recognized as affecting children aged 6 months to <4 years. In our series, 73.8% of cases of PSAHO occurred in patients in this age group, and *K. kingae* was the primary pathogen identified. Analogously, an earlier study by Spyropoulou et al. mentioned that approximately 85% of cases of PSAHO affected patients younger than 4 years and that *K. kingae* was the only microorganism cultured positively in their series [12]. In the infantile form, the clinical course of PSAHO can easily be explained by *K. kingae*’s naturally low virulence. Indeed, osteoarticular infections caused by *K. kingae* are characterized by mild-to-moderate clinical and biological inflammatory responses [19,20,21,22]. The diagnosis of osteomyelitis caused by *K. kingae* is often presented late, leading to significant lytic lesions. The juvenile form mainly affects children older than 4 years, with *MSSA* constituting the main bacteriological etiology. In this situation, PSAHO should be considered the result of increased host resistance, as many children have developed the capacity to resist pyogenic pathogens and thus contain their bone infection. Colonization by *MSSA* is recognized as more frequent among younger children [23], and we now know that 20% of individuals host persistent nasal colonies and that 30% host them transiently [24]. Individuals with persistent colonies may experience *MSSA* infections that are less severe than those of non-colonized individuals [25].

Our research also validated findings that patients with PSAHO reported relatively mild osteoarticular symptoms and fever. Only 15.9% of the patients in our sample had a fever ≥ 38 °C at admission. Previous studies have also observed this low proportion, reporting from 0–42.9% (mean = 19.1%) of children with PSAHO having fever at admission [8,11,12,26,27,28,29].

The present work also confirmed the widespread idea that laboratory data rarely contribute to the diagnosis of PSAHO. Blood tests generally show a slightly increased ESR (69.2% of cases), whereas WBC counts and CRP levels are likely to be normal or only slightly higher than expected. In fact, abnormal WBC counts were recorded in only 18.9% of cases in the present series, with abnormal CRP levels in 39.6%. Several previous studies had already made this observation, reporting abnormal ESRs in from 57.1–81.3% of cases [8,10,11,28,29], with abnormal WBC counts and CRP values in 0–31.6% [10,11,28,29] and 12.5–39.8% of cases [8,10,11,28,29], respectively. The lack of a significant systemic response to infection may simply reflect an adequate local host response to a pathogen of low virulence, such as *K. kingae*, or the presence of high-level adaptive immunity that makes subsequent infections milder. Moreover, the present study demonstrated that using the usual model of classical predictive values for osteoarticular infection (T ≥ 38 °C; WBC > 12,000/mm^3^; ESR > 20 mm/h; CRP > 10 mg/L) proved to be of very little use for identifying PSAHO.

Our research also highlighted that standard bacteriological analyses are not very effective at recognizing the pathogen responsible for PSAHO. Only 4.2% of blood cultures and 25.7% of bone aspirations were positive in our series, revealing poor performance of standard basic cultures. This observation had already been established in most of the major case series on this issue. In this regard, previous series have demonstrated that only 0–12.5% of blood cultures have been able to detect the pathogen responsible for PSAHO [7,8,9,10,11,27,28,30], with 0–75% of bone aspirate samples resulting in a bacteriological diagnosis [7,8,9,10,11,27,28,30]. The present study led us inexorably to the conclusion that appropriate NAATs drastically improve detection rates for the pathogens responsible for PSAHO. Using NAATs in combination with standard cultures has shown that the presence of a pathogen can be reasonably suspected in more than 60–65% of children with PSAHO [12,29], whereas standard culture techniques alone succeed in detecting a pathogen in only 40% of cases [7,9,11,27,28,30]. It is thus reasonable to expect that the more widespread use of classic NAATs and the advent of metagenomic next-generation sequencing technology will raise the proportion of bacterial infections detected over the next few years.

Alongside progress in recognizing the bacterial etiology of PSAHO, it is essential to emphasize the importance of MRI in its early detection. We observed that nearly 50% of lesions were undetectable using radiology but were visible on MRI. This is important because reducing diagnostic delays can prevent PSAHO from developing large lytic lesions. MRI is also the best imaging technology for assessing injuries to both growth cartilage and articular cartilage [16,17], and it is thus strongly recommended that children presenting with PSAHO undergo MRI.

Our research also suggested that most PSAHO in children follow a benign course and, consequently, treatment should be adjusted to the infection’s true clinical impact. Paradoxically, one of the main difficulties in managing subacute osteomyelitis is establishing a precise diagnosis [31,32]. Due to its very heterogeneous radiological presentation, several other benign and even malignant conditions must be considered in the differential diagnosis, including eosinophilic granuloma, osteoid osteoma, chondroblastoma, tuberculosis in endemic regions, fungal infection, osteosarcoma, Ewing’s sarcoma, leukemia, or round-cell tumors [32]. Thus, the current recommended treatment for subacute osteomyelitis with radiographic evidence of lucent lesions or nidus is curettage, biopsy, and culture, followed by antibiotics [32]. However, many authors have suggested that antibiotics alone may be adequate and that surgery should only be considered for “aggressive lesions” and those that do not respond to antibiotics [2,9].

Our study had some limitations. Its retrospective nature increased the proportion of missing data and patients lost to follow-up. In many cases, the bacteriological diagnosis remains incomplete, especially for cases where a diagnostic puncture was not indicated or was even harmful. As a result, in many situations, the involvement of *K. kingae* remained at the level of strong suspicion based on PCR performed on the oropharyngeal swab, without definitive proof. In other situations (3 cases), bacteriological investigations of samples revealed S. epidermidis, an organism that is often considered a blood culture contaminant [33,34,35]. However, recent reports suggested that it could be a rare and unexpected cause of acute or chronic osteomyelitis, even in the absence of recent surgical intervention or orthopedic implants. Since, in more than half of cases, the bacteriological diagnosis was made through PCR assays, this work lacks crucial information on the pathogens’ antimicrobial resistance. Finally, one might suspect that some cases without obvious lesions on radiographs were probably not investigated using MRI and were thus missed in the early years covered by the study. The descriptive material examined nevertheless provided lots of information about the incidence, characteristics, clinical presentations, biological impact, and bacteriological etiologies of PSAHO. Our results should be confirmed and enriched via future multicenter studies that examine larger numbers of patients.

## 5. Conclusions

Pediatric PSAHO is a distinct form of osteomyelitis characterized by an insidious onset, mild symptoms, and few, if any, systemic reactions and anomalies in laboratory data. Two forms appear distinguishable based on patient age and bacteriological etiology. The infantile form affects children aged from 6 months to <4 years, and most of the time, it is due to *K. kingae*. The juvenile form affects children aged 4 years or older, and *S. aureus* is the main bacteriological cause. Although hemocultures and classic cultures of bone aspirates usually fail to isolate the causative pathogen, appropriate nucleic acid amplification tests significantly improve the detection rate of microorganisms responsible for PSAHO. Finally, investigations using MRI may considerably improve the identification of PSAHO lesions before a lytic bone lesion becomes visible on plain radiographs.

## Figures and Tables

**Table 1:** Distribution of clinical and laboratory parameters.

	Temperature (°C)	WBC (1000/μL)	Platelets (1000/μL)	CRP (mg/L)	ESR (mm/h)
N	107	106	106	106	91
Missing	0	1	1	1	16
Mean	37	9.48	403.6	14.57	30.23
Median	37	9.2	409.5	5	28
Standard deviation	0.68	2.87	117.29	20.47	16.83
Minimum	35.8	1.6	127	0.4	4
Maximum	39.6	19	755	140	95

WBC is white blood cell, CRP is C-reactive protein, and ESR is erythrocyte sedimentation rate.

**Table 2:** Distribution of clinical and biological results regarding abnormal cut-offs.

	Value	n	% of Total
Temperature at admission	<38 °C	90	84.1
	≥38 °C	17	15.9
WBC count	≤12,000 cells/μL	86	81.1
	>12,000 cells/μL	20	18.9
CRP level	≤10 mg/L	64	60.4
	>10 mg/L	42	39.6
ESR level	≤20 mm/h	28	30.8
	>20 mm/h	63	69.2
Platelet count	≤400,000 platelets/μL	49	46.2
	>400,000 platelets/μL	57	53.8

‘n’ indicates the number of patients with available data for each specific variable: Temperature at admission is in degrees (n = 107), WBC is white blood cell (n = 106), CRP is C-reactive protein (n = 106), and ESR is erythrocyte sedimentation rate (n = 91) and platelet count (n = 106).

**Table 3:** Results of bacteriological investigations.

	Blood Culture	Bone Sample Culture	Blood *K. kingae* PCR	Sample Broad Range PCR	Sample *K. kingae* PCR	Oropharyngeal Swab PCR
N	72	70	17	17	33	47
Missing cases	35	37	90	90	74	60
Positive cases	3	18	3	4	27	39
Positive rate	4.2%	25.7%	17.7%	23.5%	81.8%	83.0%

PCR is the polymerase chain reaction.

**Table 4:** Pathogens responsible for subacute osteomyelitis (n = 107).

Pathogens	N	% of Total
*Kingella kingae*	29	27.1%
No pathogen detected	26	24.3%
Suspected *K. kingae*	32	29.9%
*Methicillin-susceptible Staphylococcus aureus*	9	8.4%
*Staphylococcus epidermis*	3	2.8%
*Streptococcus pneumoniae*	3	2.8%
*Mycobacterium tuberculosis*	1	0.93%
*Moraxella lacunata*	1	0.93%
*Streptococcus agalactiae B*	1	0.93%
Gram (-)	1	0.93%
No investigation	1	0.93%

## Data Availability

The data supporting the findings of this study are not openly available due to sensitivity concerns. They are accessible from the corresponding author upon reasonable request. The data are stored in a controlled-access repository at Geneva University Hospitals, Switzerland.

## Abbreviations

The following abbreviations are used in this manuscript:

PSAHO	Primary subacute hematogenous osteomyelitis
NAATs	Nucleic acid amplification tests
*K. kingae*	*Kingella kingae*
*S. aureus*	*Staphylococcus aureus*
*MSSA*	*Methicillin-susceptible Staphylococcus aureus*
MRI	Magnetic resonance imaging
WBC	White blood cell count
ESR	Erythrocyte sedimentation rate
CRP	C-reactive protein
PCR	Polymerase chain reaction
